# Shiga Toxin–Producing *Escherichia coli* O157, England and Wales, 1983–2012

**DOI:** 10.3201/eid2204.151485

**Published:** 2016-04

**Authors:** Natalie L. Adams, Lisa Byrne, Geraldine A. Smith, Richard Elson, John P. Harris, Roland Salmon, Robert Smith, Sarah J. O’Brien, Goutam K. Adak, Claire Jenkins

**Affiliations:** Public Health England, London, UK (N.L. Adams, L. Byrne, G.A. Smith, R. Elson, J.P. Harris, G.K. Adak, C. Jenkins);; National Institute for Health Research Health Protection Research Unit in Gastrointestinal Infections, Liverpool, UK (N.L. Adams, J.P. Harris, S.J. O’Brien, G.K. Adak);; National Institute for Health Research Health Protection Research Unit in Gastrointestinal Infections, Colindale, UK (N.L. Adams, G.K. Adak, C. Jenkins);; Public Health Wales, Cardiff, UK (R. Salmon, R. Smith)

**Keywords:** Shiga-toxigenic Escherichia coli, Escherichia coli O157, STEC, foodborne diseases, zoonoses, bacteria, gastrointestinal diseases, communicable diseases, England, Wales, enteric infections

## Abstract

We evaluated clinical Shiga toxin–producing *Escherichia coli* O157 infections in England and Wales during 1983–2012 to describe changes in microbiological and surveillance methods. A strain replacement event was captured; phage type (PT) 2 decreased to account for just 3% of cases by 2012, whereas PT8 and PT21/28 strains concurrently emerged, constituting almost two thirds of cases by 2012. Despite interventions to control and reduce transmission, incidence remained constant. However, sources of infection changed over time; outbreaks caused by contaminated meat and milk declined, suggesting that interventions aimed at reducing meat cross-contamination were effective. Petting farm and school and nursery outbreaks increased, suggesting the emergence of other modes of transmission and potentially contributing to the sustained incidence over time. Studies assessing interventions and consideration of policies and guidance should be undertaken to reduce Shiga toxin–producing *E. coli* O157 infections in England and Wales in line with the latest epidemiologic findings.

## Abstract 2

Although incidence remained constant, outbreaks from contaminated meat and milk declined and those from petting farms and schools and nurseries increased.

Shiga toxin–producing *Escherichia coli* (STEC) serogroup O157 emerged as a pathogen of public health concern during the early 1980s and was first isolated in the United Kingdom in July 1983 (Figure 1) from 3 cases linked to an outbreak of hemolytic uremic syndrome (HUS) (*1*). After this emergence, the Gastrointestinal Bacteria Reference Unit (GBRU), Public Health England (PHE) (then the Public Health Laboratory Service), reviewed a large archive of isolates and concluded that, before 1983, STEC O157 was not a major cause of gastrointestinal disease in England and Wales (*2*).

**Figure 1:**
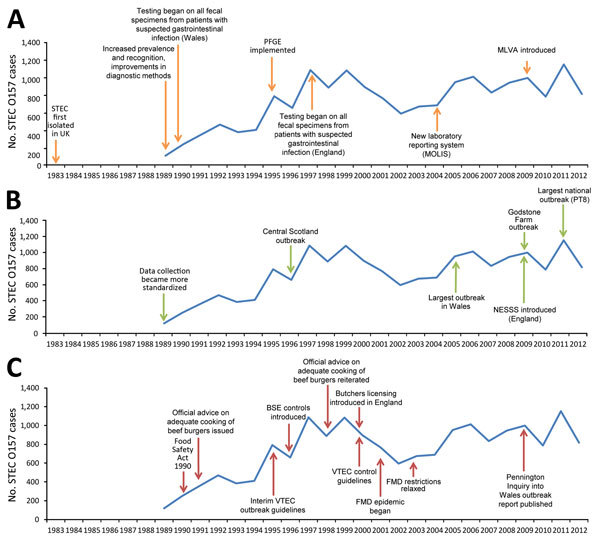
Timeline of key events influencing the epidemiology (A), microbiology (B), and guidance and control (C) of STEC O157, England and Wales, 1983–2012. Numbers before 1989 are available only as an aggregate for that period and therefore cannot be presented by year. BSE, bovine spongiform encephalopathy; FMD, foot and mouth disease; MLVA, multilocus variable-number tandem-repeat analysis; MOLIS, Modular Open Laboratory Information System; NESSS, National Enhanced Surveillance Scheme for STEC; PFGE, pulsed-field gel electrophoresis; PT, phage type; STEC, Shiga toxin–producing *Escherichia coli*; VTEC, verocytotoxin–producing *E. coli*.

Despite being relatively rare in comparison with other gastrointestinal infections, STEC O157 is of public health concern because of its potential for severity. Symptoms of infection include abdominal cramps, nausea, and bloody diarrhea. In 5%–14% of cases, infection leads to HUS, a severe and potentially fatal systemic condition primarily affecting the kidneys (*3*). The primary STEC virulence factor is Shiga toxin (Stx), which targets cells expressing the glycolipid globotriaosylceramide, disrupting host protein synthesis and causing apoptotic cell death (*4*). Children and elderly persons are most susceptible to severe illness, and HUS is recognized as the most common cause of acute renal failure among children in the United Kingdom (*5*).

Cattle and other ruminants are natural reservoirs for STEC O157, and transmission to humans occurs through direct or indirect contact with the animals or their feces or through ingestion of contaminated food or water. A low infectious dose and propensity for person-to-person spread means transmission in households and closed settings such as schools is common (*6*,*7*), as is the potential for large outbreaks (*8*–*12*). We describe changes in the epidemiology of STEC O157 in England and Wales during a 30-year period (1983–2012) against a background of changing microbiological and surveillance methods over time.

## Methods

### Case Ascertainment, 1983–2012

Beginning in 1983, only fecal specimens from patients with HUS or hemorrhagic colitis were referred for STEC O157 testing; before 1989, few specimens were referred for testing. Beginning in 1997 in England and Wales, referral for testing was extended to all patients with symptoms of gastrointestinal infection, including vomiting, diarrhea, or bloody feces.

### Microbiology Methods, 1983–2012

GBRU provides the national reference service for STEC in England and Wales. Beginning in 1983, individual colonies were tested for toxin production by using the verocytotoxin cell assay, and positive colonies were identified biochemically and serotyped (*13*). In 1987 at GBRU, the verocytotoxin cell assay was replaced with a molecular probe assay to detect the *stx* gene (*14*) and sorbitol MacConkey culture medium. Later, sorbitol MacConkey culture medium containing cefixime and tellurite was developed, facilitating isolation of STEC O157 from fecal specimens, but testing was not implemented for all patients with symptoms of gastrointestinal infection in all local hospital laboratories until 1997 (*15*). Isolates of *E. coli* O157 identified locally are sent for confirmation and typing at GBRU.

Detection and confirmation of STEC at GBRU includes biochemical identification and serotyping of bacterial isolates. Since 1989, strains belonging to *E. coli* O157 have been further differentiated by using a phage typing scheme developed in Canada (*16*). Retrospective phage typing was undertaken for all viable strains collected before 1989. During 1994–2011, detection of *stx1* or *stx2* used a block-based PCR (*17*), which was replaced in 2012 with real-time PCR targeting *stx1* or *stx2* and the intimin (*eae*) gene, associated with intimate attachment of the bacteria to the host gut mucosa (*18*).

### Data Collection Methods, 1983–2012

The amount of epidemiologic and microbiological data increased during the study period. During 1983–2003, a dedicated laboratory database was used to record patient and microbiological data. In 2004, a new laboratory reporting system, Modular Open Laboratory Information System, was implemented (Figure 1). These laboratory databases captured microbiological results and demographic details of cases, as well as limited epidemiologic data (HUS diagnosis, outbreak association, recent history of foreign travel).

In January 2009, PHE introduced the National Enhanced Surveillance Scheme for STEC (Figure 1) (*19*). This scheme captured epidemiologic information through standardized questionnaires administered to all persons with STEC and linked to microbiological data in the Modular Open Laboratory Information System.

Detection of outbreaks relied on detecting unusual increases in STEC activity or reporting of shared exposures among cases of the same phage type (PT). Outbreaks were recorded on paper before 1992. In 1992, PHE began standardized surveillance of outbreaks of gastrointestinal disease where >2 persons with the same infection are linked, or probably linked, to the same source. In brief, local PHE units report standardized epidemiologic data on all outbreaks of gastrointestinal diseases, including source of infection and microbiological data.

### Data Analyses

Data on STEC O157 patients in England and Wales were analyzed in 3 time periods, 1983–1988, 1989–1996, and 1997–2012, to account for periods of differing case ascertainment and data collection. Case numbers for 1989–1996 were small and represent biased sampling toward severe STEC O157 infections therefore calculation of incidence and interpretation of trends would be meaningless, and these were calculated only for 1997–2012.

We performed descriptive analyses in Microsoft Excel 2010 (Microsoft Corporation, Redmond, WA, USA). Crude incidence rates were calculated by using the Office of National Statistics mid-year population estimates (*20*). Crude incidence rate ratios (RR) and 95% CIs were calculated in Stata version 13.0 (StataCorp LP, College Station, TX, USA) for comparison among groups.

## Results

### Microbiology of STEC O157, 1983–2012

In England and Wales during 1983–1988, a total of 279 patients were infected with STEC O157, including 110 from 3 outbreaks. Of the 169 non–outbreak-related isolates, 155 were retrospectively phage typed; the most common types were PT2 (49 [31.6%] cases), PT1 (38 [24.5%]), and PT49 (22 [14.2%]).

**Figure 2:**
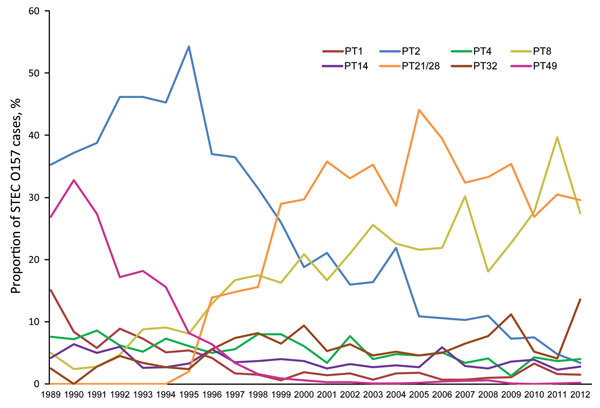
Proportions of common phage types (PTs) of Shiga toxin–producing *Escherichia coli* O157 identified, England and Wales, 1989–2012.

During 1989–1996, the number of cases increased (3,448 total cases), and the proportions of common PTs changed annually (Figure 2). In 1996, a new PT was designated PT21/28 after reexamination of the lysis profiles of PT21 and PT28 isolates (*21*). By 1996, PT2 (244 [37%] isolates), PT8 (85 [12.9%] isolates), and PT21/28 (92 [13.9%] isolates) were the most common PTs, and the proportion of PT1 (28 [4.2%] isolates) and PT49 (42 [6.4%] isolates) had declined.

During 1997–2012, the decline in these PTs continued, and PT1 and PT49 were rarely observed. PT2 also declined to just 28 (3.4%) isolates by 2012 from a peak of 430 (54.3%) isolates in 1995, a significant decrease for this period (p<0.001) (Figure 2). Concurrently, numbers of PT21/28 rapidly increased, accounting for 420 (44.1%) cases by 2005, a significant increase for the period (p<0.001), and thereafter remaining the most frequently detected PT (Figure 2). PT8 also increased significantly, from 182 (16.7%) to 225 (27.5%) of cases by 2012 (p<0.001).

Strains encoding Stx1 only were rare (81 [0.6%] isolates), and most (45 [55.6%] isolates) were PT8. Strains encoding Stx2 only were most frequent (10,182 [71.8%] isolates), followed by Stx1+2 (3,921 [27.6%] isolates). Stx type and PT are interrelated; most PT8 strains (3,040 [93.2%] isolates) possessed *stx1+2*, whereas PT2 and PT21/28 usually possessed *stx2* only (2,100 [92.8%] and 4,340 [99.7%] isolates, respectively).

### Epidemiology of STEC O157, 1997–2012

#### Case Numbers and Crude Incidence

**Figure 3:**
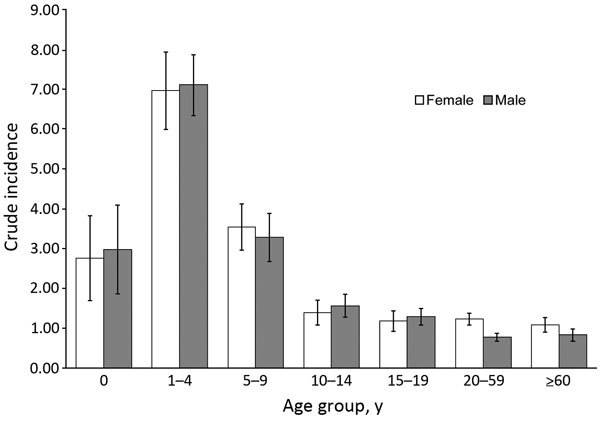
Crude incidence (cases per 100,000 person-years) of Shiga toxin–producing *Escherichia coli* O157, by patient age group and sex, England and Wales, 1997–2012. Error bars indicate 95% CIs.

A total of 14,184 laboratory-confirmed STEC O157 cases were identified in England and Wales; the mean was 887 (95% CI 802–972) cases per year. Crude incidence was 1.65 (95% CI 1.49–1.81) cases/100,000 person-years but varied by year, geography, and patient age and sex (Figure 3). Identifiable peaks in case numbers corresponded to reported outbreaks (Figure 1; Table). Crude incidence decreased from 1999, reaching its lowest in 2002 (1.1 cases/100,000 person-years [595 cases]), but returned to previous levels in 2005 and was sustained thereafter.

**Table:** Reported outbreaks of STEC O157, England and Wales, 1983–2012*

Year	No. outbreaks	PT (no. outbreaks)	No. STEC cases	No. HUS cases	Implicated vehicle (no. outbreaks)
1983	1	2 (1)	Unknown	35	Unknown (1)
1984	0	–	–	–	–
1985	1	2 (1)	49	0	Raw potatoes (1)
1986	0	–	–	–	–
1987	1	2 (1)	26	0	Turkey roll (1)
1988	0	–	–	–	–
1989	1	49 (1)	8	1	Unknown (1)
1990	3	2 (1), 4 (1), 14 (1)	18	3	Unknown (1)
1991	4	14 (1), 31 (1), 49 (2)	56	8	Yoghurt (1), beef burger (1), person to person (1), unknown (1)
1992	6	1 (1), 2 (5)	82	5	Cooked meat (1), unknown (5)
1993	7	2 (3), 28 (1), 49 (3)	65	13	Beef (1), beef burger (1), raw milk (1), unknown (4)
1994	6	2 (1), 4 (1), 49 (2), RDNC (1), unknown (1)	37	6	Direct animal contact (2), person to person (1), unknown (3)
1995	11	1 (1), 2 (7), 49 (2), RDNC (1)	141	11	Salad (1), meat (3), person to person (4), unknown foodborne (1), unknown (2)
1996	17	1 (2), 2 (5), 8 (3), 12 (1), 21/28 (4), 32 (1), unknown (1)	98	2	Animal contact (1), person to person (1), milk (2), meat (4), mixed food (2), raw vegetables (1), unknown foodborne (3), unknown (3)
1997	25	2 (13), 8 (5), 49 (1), 21/28 (6)	163	9	Animal contact (6), cheese (1), milk (2), salad (1), person to person (5), water (1), unknown foodborne (4), unknown (5)
1998	20	2 (7), 4 (1), 8 (3), 14 (2), 21 (1), 32 (1), 38 (1), 21/28 (3), unknown (1)	89	0	Animal contact (2), person to person (5), meat (2), cream (1), milk (1), unknown foodborne, water (2), unknown (6)
1999	19	2 (3), 4 (2), 8 (2), 31 (1), 33 (1), 43 (1), 21/28 (9)	236	15	Animal contact (3), person to person (3), water (1), meat (3), milk (3), cheese (1), unknown foodborne (2), unknown (3)
2000	23	1 (1), 2(2), 8 (4), 14 (1), 32 (2), 21/28 (10), RDNC (1), unknown (2)	180	11	Animal contact (3), meat (5), milk (3), person to person (7), unknown foodborne (1), unknown (4)
2001	18	2 (3), 8 (1), 21/28 (13), unknown (1)	127	6	Animal contact (3), person to person (9), water (2), meat (2), unknown foodborne (1), unknown (1)
2002	9	2 (2), 4 (2), 34 (1), 21/28 (3), RDNC (1)	73	2	Animal contact (1), milk (1), salad (1), person to person (5), water (1)
2003	10	2 (1), 8 (2), 50 (1), 21/28 (3), RDNC (1), unknown (1)	82	0	Animal contact (5), meat (1), person to person (3), unknown (1)
2004	15	2 (5), 21/28 (9), unknown (1)	92	4	Animal contact (4), meat (2), person to person (3), water (3), unknown (3)
2005	15	2 (1), 8 (1), 33 (1), 21/28 (10), unknown (2)	233	6	Animal contact (3), meat (1), unknown foodborne (2), person to person (5), unknown (4)
2006	12	2 (1), 8 (4), 28/28 (6), 4 and 21/28 (1)	151	0	Animal contact (1), meat (1), unknown foodborne (3), person to person (5), water (2)
2007	14	2 (5), 4 (1), 8 (2), 21/28 (4), RDNC (1), 32 and 8 (1)	117	3	Animal contact (2), meat (1), unknown foodborne (1), person to person (9), unknown (1)
2008	17	2 (2), 8 (1), 33 (3), 34 (1), 21/28 (7), RDNC (1), mixed unknown (1), unknown (1)	132	4	Animal contact (4), unknown foodborne (4), person to person (6), water (1), unknown (2)
2009	25	2 (5), 8 (4), 14 (1), 32 (2), 21/28 (9), 2 and 8 (1), 2 and 54 (1), 31 and 42 (1), 34 and 54 (1)	280	30	Animal contact (11), meat (2), unknown foodborne (6), person to person (2), laboratory acquired (1), unknown (3)
2010	17	1 (2), 2 (1), 4 (2), 8 (3), 14 (1), 21/28 (5), 1 and 21/28 (1), 21/28 and 31 (1), 21/28 & RDNC (1)	100	3	Animal contact (7), unknown foodborne (1), person to person (5), unknown (4)
2011	20	2 (2), 8 (3), 34, 51, 21/28 (12), RDNC	369	7	Animal contact (1), sandwiches (2), crab (1), leeks (1), ice cream (1), unknown foodborne (4), laboratory acquired (1), person to person (6), unknown (3)
2012	18	2, 4 (2), 8 (4), 32, 54, 21/28 (8), 32	103	5	Animal contact (4), environmental (2), meat (2), unknown foodborne (2), person to person (7), unknown (1)
Total	335		3,107	189	

*Gastrointestinal Bacteria Reference Unit, Public Health England, and Gastrointestinal Infections Department, Public Health England in-house data. HUS, hemolytic uremic syndrome; PT, phage type; RDNC, reacts but does not conform; STEC, Shiga toxin–producing *Escherichia coli*.

STEC O157 infections demonstrated a distinct seasonality. Cases began to increase in April and declined beginning in September (data not shown).

#### Patient Age and Sex

Patient age was reported for 13,015 (91.8%) cases. Children <15 years of age constituted 5,867 (45.1%) cases; the greatest proportion (2,970 [22.8%] cases) occurred among those 1–4 years of age. Crude incidence decreased with increasing age; incidence was lowest for persons >60 years of age (0.98 [95% CI 0.82–1.12] cases/100,000 person-years) (Figure 3). Crude incidence was significantly higher for children 1–4 years of age (7.21 [95% CI 6.34–8.04] cases/100,000 person-years) than for those 20–59 years of age (RR 7.16, p<0.001) and >60 years of age (RR 7.36, p<0.001).

Sex was reported for 13,947 (98.3%) patients. Female patients accounted for 7,717 (55.3%) cases, and crude incidence was significantly higher for female than for male patients (RR 1.19, p<0.001; 1.76 [95% CI 1.59–1.93] cases/100,000 person-years , vs. 1.48 [95% CI 1.34–1.62] cases/100,000 person-years). Age and sex were reported for 12,848 (90.6%) patients. Sex disparity was highest for those 20–59 years of age (RR 1.60 for women vs. men; p<0.001).

The proportion of Stx2-only strains decreased with increasing age. Most (288 [81.8%]) children 1–4 years of age were infected with strains carrying Stx2 only, compared with 1,352 (65.4%) of persons >60 years of age (p<0.001). In parallel, the proportion of Stx1+2 profiles increased with age; 548 (16.1%) 1–4-year-olds were reported to have Stx1+2, compared with 698 (33.7%) of persons >60 years of age (p<0.001). We found no differences in sex by PT or Stx.

#### Geography

Annual crude incidence was highest in Cumbria and Lancashire (North West England) (3.70 [95% CI 2.70–4.70] cases/100,000 person-years), followed by Yorkshire and Humber (North East England, 2.75 [95% CI 2.37–3.13] cases/100,000 person-years) and Devon, Cornwall, and Somerset (South West England, 2.71 [95% CI 2.35–3.07] cases/100,000 person-years), whereas annual crude incidence was lowest in London (0.99 [95% CI 0.83–1.14] cases/100,000 person-years). Cases were almost 4 times more likely to be reported in Cumbria and Lancashire (RR 3.72) than in London (p<0.001). Within areas, crude incidence remained stable over time, other than peaks associated with outbreaks. We found no notable differences by geography in age, sex, PT, or seasonality.

### Outbreaks, 1983–2012

During 1983–2012, a total of 335 outbreaks were reported, ranging from 0 to 25 outbreaks annually (Table). These outbreaks constituted 3,107 (17.4%) cases (median 5 cases, range 2–257 cases).

Large outbreaks caused peaks in annual crude incidence (Figure 1). For example, in 1995, eleven outbreaks comprising 141 cases occurred (Table), including a large nursery outbreak in Wales affecting 49 children (*6*). In 1999, nineteen outbreaks (236 cases) occurred, causing incidence to peak. Nine were attributed to contaminated food vehicles, including 3 caused by milk pasteurization failures, 1 affecting 88 persons (*22*). Outbreaks caused by postpasteurization contamination of milk also occurred in 2000 and 2002, as did 2 outbreaks associated with drinking raw milk in 2000, but no milk-related outbreaks were observed during the remainder of the study period.

Food vehicles contributed the highest number of outbreaks (101 [30.3%]) and outbreak cases (1,418 [45.9%]) (Table). These outbreaks included 38 attributed to eating contaminated meat; 16 to eating undercooked meat, such as burgers at barbecues; and 22 to cross-contamination of cooked meats. The cross-contamination outbreaks were larger; the largest meat-related outbreak occurred in Wales when meat from a butcher supplied to institutions infected 118 persons with STEC O157 in 2005 (*12*). After that, meat-related outbreaks were infrequent; 7 meat-related outbreaks (compared with 31 before this outbreak) were reported in the subsequent 7 years.

The first implicated food vehicle in this study was raw potatoes in a 1985 outbreak, and outbreaks associated with eating vegetables were reported throughout the years. The largest national outbreak in Great Britain (252 cases) caused by STEC O157 PT8, linked to handling raw leeks and potatoes, was reported in 2011 and led to the highest incidence during the period (Figure 1) (*23*).

Person-to-person spread in institutional settings accounted for 29.1% of outbreaks and more than one quarter of outbreak cases (825 cases). Twenty-six outbreaks occurred in institutional settings: care homes (16 outbreaks), prisons (4 outbreaks), and hospitals (6 outbreaks). No outbreaks in these settings occurred after 2007. Seventy-two outbreaks, which resulted in 808 cases, occurred in childcare facilities. Each year, 1–7 outbreaks in child-care facilities occurred, but outbreaks increased in frequency in later years; during 1983–2003, a total of 25 outbreaks (313 cases) were reported, whereas 47 outbreaks (495 cases) were reported in the subsequent 9 years.

Direct or indirect contact with animals through the environment accounted for 22.4% of outbreaks and 17.3% of patients linked to outbreaks. The number of petting farm outbreaks increased during the study period. During 1983–2002, a total of 12 petting farm outbreaks were reported; during 2003–2012, a total of 31 outbreaks on petting farms were reported, including, in September 2009, the largest reported farm outbreak, which affected 93 persons (*9*).

Most outbreaks were caused by the most frequently detected STEC O157 PTs, including PT21/28 (117 [34.9%] outbreaks), PT2 (79 [23.6%] outbreaks), and PT8 (42 [12.5%] outbreaks). In accordance with the general trends in PT, PT2 outbreaks declined over time, whereas PT8 and PT21/28 outbreaks increased. For outbreaks attributed to contact with animals or their environments, almost half (28 [47.5%] outbreaks) were caused by PT21/28 strains, a further 16 (27.1%) by PT2 strains, and only 4 (6.8%) by PT8 strains. Ten outbreaks attributed to contaminated water were caused by PT2 (5 outbreaks), PT21/28 (4 outbreaks), and PT4 (1 outbreaks); none were caused by PT8. In foodborne outbreaks, 25 (28.1%) were caused by PT2, 32 (36.1%) by PT21/28, and 20 (22.5%) by PT8.

## Discussion

Our review provides a historical perspective contributing to the evidence of the evolving epidemiology of STEC O157. The data capture a strain replacement event showing the dramatic decline in PT2 and the increase and dominance of PT8 and PT21/28. Outbreak settings and vehicles also changed during the study period; prison, hospital, and care-home outbreaks decreased, and outbreaks in childcare facilities increased. Additionally, outbreaks associated with meat and milk decreased, and outbreaks attributed to petting farms increased. These data support previous reports that PT21/28 is indigenous to Great Britain and PT8 is largely imported, because most PT8 outbreaks were foodborne and a greater proportion of PT21/28 were attributed to environmental or animal contact (*19*,*21*).

The reasons for the decline in STEC incidence during 2000–2004 are unknown and cannot be attributed to any particular event or intervention, although several possible explanations exist. After a large STEC O157 outbreak in central Scotland in 1996 (*8*), specific interventions were implemented throughout the entire United Kingdom in catering, retail, and meat hygiene sectors to reduce the risk for infection. These included butchers’ licensing, legislation, and enforcement of Hazard Analysis and Critical Control Point systems; amendment of the Food Standards Agency Code of Practice; and introduction of the Clean Livestock Policy, which aimed to reduce contamination by feces or mud on the coats and fleeces of animals for slaughter (*24*). The effectiveness of these policies was apparent through the shift in causes of outbreaks presented in this study; after their implementation, outbreaks caused by cross-contamination from raw meat clearly declined.

Why the decline in STEC incidence was not sustained beyond 2004 is unclear; however, declining numbers were observed in the United States in 2003 and 2004, followed by increases beginning in 2005. The decline coincided with industry measures aimed at reducing contamination of ground beef; however, as in the United Kingdom, the reason for the subsequent increase is unknown (*25*). Apparent changes in sources and outbreak settings might indicate changes in food vehicles or transmission routes among all cases, and although earlier interventions successfully controlled transmission of STEC infection, other effective transmission routes have taken hold in more recent years. Also, in this study, outbreak detection relied on the classic epidemiologic triad of person, place, and time, along with PT. Any outbreaks dispersed over time, or of a common PT, might have gone undetected. Data collected on outbreaks and sources—and therefore trends—will be incomplete.

Farming methods and destruction of animal populations changed considerably during the study period after concerns about bovine spongiform encephalopathy in 1996 and foot and mouth disease in 2001. The decline of PT1, PT2, and PT49, and the corresponding emergence of PT21/28, was mirrored in Scotland (*26*) and suggests a strain replacement event. The destruction and restocking of UK cattle herds after concerns about bovine spongiform encephalopathy and foot and mouth disease might have been a causative factor. In Ireland, PT32 is the most commonly reported PT (*27*); PT21/28 is rarely detected outside the British Isles (*19*).

Improvements in data collection during our study led to increased ascertainment of epidemiologic data during the 30-year period alongside important developments in microbiological methods. Thus, the sustained incidence of infection could be a surveillance artifact, masking the success of interventions through increasing case ascertainment, a potential bias when datasets spanning many years, such as this one, are analyzed. In England and Wales, although surveillance of clinical STEC infections is routine, no surveillance programs are ongoing to monitor the prevalence of STEC in cattle or other animals. Efforts by the agricultural, veterinary, and food industries to monitor STEC incidence and strain types would inform the success of interventions and provide insight into the ecology of the pathogen. However, STEC rarely causes disease in animals, and funding is limited for such programs in England and Wales. In Europe, current monitoring information is generated from outbreak investigations and ad hoc studies skewed toward foodborne transmission of STEC O157 and might be limited in assessing the role of environmental transmission.

As described previously, infection is highest in children and females (*5*,*19*,*28*). Children 1–4 years of age had 7 times the risk of persons >60 years of age, probably because of a complex interplay of various factors, such as host immunity or reporting artifacts, with children more likely to seek care at healthcare settings (*29*). Additionally, the propensity for household transmission of STEC O157 (*30*) might be exacerbated by children having poorer hygiene practices that increase exposure to STEC O157 from the environment (*19*), and the potential for prolonged excretion in children (*7*). Children were more often infected with STEC O157 Stx2-only strains, associated with more severe disease (*4*,*5*,*21*,*31*), which might in part explain why cases occurred more often in children, because they were more likely to require care at healthcare settings. The higher crude incidence rates for female than for male patients has been reported previously (*19*,*28*); the reasons are unknown but might reflect biologic host factors, differences in health-seeking behavior, or other behaviors placing women at increased risk for infection, such as having contact with children or being primary household food handlers (*19*,*32*).

In our review, crude incidence was higher in the north and southwest than in the central and southeastern areas of England. Crude incidence in Scotland is consistently higher still (*19*). Previous studies have described such geographic variation and demonstrated that differences reflect differences in weather, land use, or environmental exposure between persons living in or visiting rural areas and those in urban areas, fitting with environmental transmission of STEC O157 (*19*,*33*).

Our 30-year review captures the emergence of a clinically significant zoonotic pathogen in a well-characterized population sample and documents the effectiveness of improvements in epidemiologic and microbiological methods on ascertaining STEC O157. However, despite interventions that successfully shifted outbreak settings, these organisms persist in causing illness in England and Wales, and the crude incidence of STEC O157 has remained relatively stable. Robust studies are required to assess the effectiveness of interventions, which currently remain unclear, and to consider future policies and guidance to reduce STEC O157 infection in England and Wales in the context of the complex interaction between the organism, reservoir, food chain, and transmission pathway.

Technical Appendix Reported outbreaks of Shiga toxin–producing *Escherichia coli* O157, England and Wales, 1983–2012
