# Supplementary material for: Shiga Toxin–Producing Escherichia coli O157, England and Wales, 1983–2012
Source: Emerg Infect Dis. 2016 Apr;22(4):590–7. doi: 10.3201/eid2204.151485 (PMC4806951; doi:10.3201/eid2204.151485)
Supplement: Technical Appendix — Reported outbreaks of Shiga toxin–producing Escherichia coli O157, England and Wales, 1983–2012 [file 15-1485-Techapp-s1.pdf]

Article DOI: http://dx.doi.org/10.32032/eid2204.151485

Shiga Toxin–Producing *Escherichia coli* O157, England and Wales, 1983–2012

Technical Appendix

Technical Appendix Table. Reported outbreaks of Shiga toxin–producing *Escherichia coli* O157, England and Wales, 1983–2012*

Year	No. outbreaks†	Phage type (no. outbreaks where known)	No. VTEC cases	No. HUS cases‡	Implicated vehicle§ (no. outbreaks)	References
1983	1	2 (1)	Unknown	35	Unknown (1)	(*1*)
1984	0	–	–	–	-	
1985	1	2 (1)	49	0	Raw potatoes (1)	
1986	0	–	–	–	-	
1987	1	2 (1)	26	0	Turkey roll (1)	
1988	0	–	–	–	-	
1989	1	49 (1)	8	1	Unknown (1)	
1990	3	2 (1), 4 (1), 14 (1)	18	3	Unknown (1)	(*2*)
1991	4	14 (1), 31 (1), 49 (2)	56	8	Yoghurt (1), beef burger (1), person to person (1), unknown (1)	(*2*)
1992	6	1 (1), 2 (5)	82	5	Cooked meat (1), unknown (5)	(*2–6*)
1993	7	2 (3), 28 (1), 49 (3)	65	13	Beef (1), beef burger (1), raw milk (1), unknown (4)	(*2–6*)
1994	6	2 (1), 4 (1), 49 (2), RDNC (1), unknown (1)	37	6	Direct animal contact (2), person to person (1), unknown (3)	(*2–6*)
1995	11	1 (1), 2 (7), 49 (2), RDNC (1)	141	11	Salad (1), meat (3), person to person (4), unknown foodborne (1), unknown (2)	(*2,4–8*)
1996	17	1 (2), 2 (5), 8 (3), 12 (1), 21/28 (4), 32 (1), unknown (1)	98	2	Animal contact (1), person to person (1), milk (2), meat (4), mixed food (2), raw vegetables (1), unknown foodborne (3), unknown (3)	(*2,4–6,9,10*)
1997	25	2 (13), 8 (5), 49 (1), 21/28 (6)	163	9	Animal contact (6), cheese (1), milk (2), salad (1), person to person (5), water (1), unknown foodborne (4), unknown (5)	(*2,5,6,11,12*)
1998	20	2 (7), 4 (1), 8 (3), 14 (2), 21 (1), 32 (1), 38 (1), 21/28 (3), unknown (1)	89	0	Animal contact (2), person to person (5), meat (2), cream (1), milk (1), unknown foodborne, water (2), unknown (6)	(*2,5,6,13,14*)
1999	19	2 (3), 4 (2), 8 (2), 31 (1), 33 (1), 43 (1), 21/28 (9)	236	15	Animal contact (3), person to person (3), water (1), meat (3), milk (3), cheese (1), unknown foodborne (2), unknown (3)	(*5,6,15–21*)
2000	23	1 (1), 2(2), 8 (4), 14 (1), 32 (2), 21/28 (10), RDNC (1), unknown (2)	180	11	Animal contact (3), meat (5), milk (3), person to person (7), unknown foodborne (1), unknown (4)	(*5,6,22*)
2001	18	2 (3), 8 (1), 21/28 (13), unknown (1)	127	6	Animal contact (3), person to person (9), water (2), meat (2), unknown foodborne (1), unknown (1)	(*23*)
2002	9	2 (2), 4 (2), 34 (1), 21/28 (3), RDNC (1)	73	2	Animal contact (1), milk (1), salad (1), person to person (5), water (1)	
2003	10	2 (1), 8 (2), 50 (1), 21/28 (3), RDNC (1), unknown (1)	82	0	Animal contact (5), meat (1), person to person (3), unknown (1)	
2004	15	2 (5), 21/28 (9), unknown (1)	92	4	Animal contact (4), meat (2), person to person (3), water (3), unknown (3)	(*24,25*)
2005	15	2 (1), 8 (1), 33 (1), 21/28 (10), unknown (2)	233	6	Animal contact (3), meat (1), unknown foodborne (2), person to person (5), unknown (4)	(*26*)
2006	12	2 (1), 8 (4), 28/28 (6), 4 & 21/28 (1)	151	0	Animal contact (1), meat (1), unknown foodborne (3), person to person (5), water (2)	
2007	14	2 (5), 4 (1), 8 (2), 21/28 (4), RDNC (1), 32 & 8 (1)	117	3	Animal contact (2), meat (1), unknown foodborne (1), person to person (9), unknown (1)	(*27,28*)

Year	No. outbreaks†	Phage type (no. outbreaks where known)	No. VTEC cases	No. HUS cases‡	Implicated vehicle§ (no. outbreaks)	References
2008	17	2 (2), 8 (1), 33 (3), 34 (1), 21/28 (7), RDNC (1), mixed unknown (1), unknown (1)	132	4	Animal contact (4), unknown foodborne (4), person to person (6), water (1), unknown (2)	
2009	25	2 (5), 8 (4), 14 (1), 32 (2), 21/28 (9), 2 & 8 (1), 2 & 54 (1), 31 & 42 (1), 34 & 54 (1)	280	30	Animal contact (11), meat (2), unknown foodborne (6), person to person (2), laboratory acquired (1), unknown (3)	(*29*,*30*)
2010	17	1 (2), 2 (1), 4 (2), 8 (3), 14 (1), 21/28 (5), 1 & 21/28 (1), 21/28 & 31 (1), 21/28 & RDNC (1)	100	3	Animal contact (7), unknown foodborne (1), person to person (5), unknown (4)	
2011	20	2 (2), 8 (3), 34, 51, 21/28 (12), RDNC	369	7	Animal contact (1), sandwiches (2), crab (1), leeks (1), ice cream (1), unknown foodborne (4), laboratory acquired (1), person to person (6), unknown (3)	(*31*,*32*)
2012	18	2, 4 (2), 8 (4), 32, 54, 21/28 (8), 32	103	5	Animal contact (4), environmental (2), meat (2), unknown foodborne (2), person to person (7), unknown (1)	
Total	335		3,107	189		

*HUS, hemolytic-uremic syndrome; STEC, Shiga toxin–producing *Escherichia coli*; VTEC, Vero cytotoxin–producing *E. coli*.
†In 1997, national protocols for the detection of VTEC in England were amended so that fecal specimens from all patients with symptoms of gastrointestinal infection, including mild diarrhea, were tested for VTEC. Before this time, specimens from persons with HUS or hemorrhagic colitis only were tested, which accounts for the apparent increase in outbreaks reported after 1996.
‡Data on cases of HUS as reported on 1) laboratory referral forms; 2) within published outbreak investigation reports, and 3) from the National Enhanced Surveillance System for VTEC in England (for 2009–2013). These data have not been collected systematically and will underestimate the true number of HUS cases and should be interpreted with caution.
§Implicated vehicles are those reported in the surveillance systems and/or publications described. Varying levels of evidence will have been available in different investigations. Data presented here reflect the conclusions of the outbreak investigations.

References

1. Taylor CM, White RH, Winterborn MH, Rowe B. Haemolytic-uraemic syndrome: clinical experience of an outbreak in the West Midlands. BMJ. 1986;292:1513–6. PubMed http://dx.doi.org/10.1136/bmj.292.6534.1513

2. Chalmers RM, Parry SM, Salmon RL, Smith RM, Willshaw GA, Cheasty T. The surveillance of Vero cytotoxin–producing *Escherichia coli* O157 in Wales, 1990 to 1998. Emerg Infect Dis. 1999;5:566–9. PubMed http://dx.doi.org/10.3201/eid0504.990422

3. Wall PG, McDonnell RJ, Adak GK, Cheasty T, Smith HR, Rowe B. General outbreaks of Vero cytotoxin producing *Escherichia coli* O157 in England and Wales from 1992 to 1994. Commun Dis Rep CDR Rev. 1996;6:R26–33. PubMed

4. Djuretic T, Wall PG, Nichols G. General outbreaks of infectious intestinal disease associated with milk and dairy products in England and Wales: 1992 to 1996. Commun Dis Rep CDR Rev. 1997;7:R41–5. PubMed

5. Gillespie IA, Adak GK, O'Brien SJ, Bolton FJ. Milkborne general outbreaks of infectious intestinal disease, England and Wales, 1992–2000. Epidemiol Infect. 2003;130:461–8. PubMed

6. Gillespie IA, O'Brien SJ, Adak GK, Cheasty T, Willshaw G. Foodborne general outbreaks of Shiga toxin–producing *Escherichia coli* O157 in England and Wales 1992–2002: where are the risks? Epidemiol Infect. 2005;133:803–8. PubMed http://dx.doi.org/10.1017/S0950268805004486

7. McDonnell RJ, Rampling A, Crook S, Cockcroft PM, Wilshaw GA, Cheasty T, et al. An outbreak of Vero cytotoxin producing *Escherichia coli* O157 infection associated with takeaway sandwiches. Commun Dis Rep CDR Rev. 1997;7:R201–5. PubMed

8. Stevenson J, Hanson S. Outbreak of *Escherichia coli* O157 phage type 2 infection associated with eating precooked meats. Commun Dis Rep CDR Rev. 1996;6:R116–8. PubMed

9. VTEC O157 infection in West Yorkshire associated with the consumption of raw milk. Commun Dis Rep CDR Wkly. 1996;6:181. PubMed

10. Clark A, Morton S, Wright P, Corkish J, Bolton FJ, Russell J. A community outbreak of Vero cytotoxin producing *Escherichia coli* O157 infection linked to a small farm dairy. Commun Dis Rep CDR Rev. 1997;7:R206–11. PubMed

11. Crampin M, Willshaw G, Hancock R, Djuretic T, Elstob C, Rouse A, et al. Outbreak of *Escherichia coli* O157 infection associated with a music festival. Eur J Clin Microbiol Infect Dis. 1999;18:286–8. PubMed http://dx.doi.org/10.1007/s100960050278

12. *Escherichia coli* O157 outbreak in Lincolnshire. Commun Dis Rep CDR Wkly. 1997;7:101. PubMed

13. Cases of *Escherichia coli* O157 infection associated with unpasteurised cream. Commun Dis Rep CDR Wkly. 1998;8:377. PubMed

14. Outbreak of Vero cytotoxin producing *Escherichia coli* O157 infection in Dorset. Commun Dis Rep CDR Wkly. 1998;8:183, 6. **PMID: 9635343**

15. Outbreak of Vero cytotoxin producing *Escherichia coli* O157 infection in north Cumbria. Commun Dis Rep CDR Wkly. 1999;9:95, 8. **PMID: 10205976**

16. VTEC O157 phage type 21/28 infection in North Cumbria: update. Commun Dis Rep CDR Wkly. 1999;9:105. PubMed

17. Goh S, Newman C, Knowles M, Bolton FJ, Hollyoak V, Richards S, et al. *E. coli* O157 phage type 21/28 outbreak in North Cumbria associated with pasteurized milk. Epidemiol Infect. 2002;129:451–7. PubMed http://dx.doi.org/10.1017/S0950268802007835

18. Harrison S, Kinra S. Outbreak of *Escherichia coli* O157 associated with a busy bathing beach. Commun Dis Public Health. 2004;7:47–50. PubMed

19. Outbreak of VTEC O157 infection at a prison in the Midlands. Commun Dis Rep CDR Wkly. 1999;9:281, 4. **PMID: 10453096**

20. *Escherichia coli* O157 associated with eating unpasteurised cheese—update. Commun Dis Rep CDR Wkly. 1999;9:131, 4. **PMID: 10333646**

21. *Escherichia coli* O157 associated with eating unpasteurised cheese. Commun Dis Rep CDR Wkly. 1999;9:113, 6. **PMID: 10230110**

22. Outbreaks of VTEC O157 infection linked to consumption of unpasteurized milk. Commun Dis Rep CDR Wkly. 2000;10:203, 6. **PMID: 10893825**

23. Rajpura A, Lamden K, Forster S, Clarke S, Cheesbrough J, Gornall S, et al. Large outbreak of infection with *Escherichia coli* O157 PT21/28 in Eccleston, Lancashire, due to cross contamination at a butcher's counter. Commun Dis Public Health. 2003;6:279–84. PubMed

24. Verma A, Bolton FJ, Fiefield D, Lamb P, Woloschin E, Smith N, et al. An outbreak of *E. coli* O157 associated with a swimming pool: an unusual vehicle of transmission. Epidemiol Infect. 2007;135:989–92. PubMed http://dx.doi.org/10.1017/S0950268807007947

25. Ihekweazu C, Barlow M, Roberts S, Christensen H, Guttridge B, Lewis D, et al. Outbreak of *E. coli* O157 infection in the south west of the UK: risks from streams crossing seaside beaches. Euro Surveill. 2006;11:128–30. PubMed

26. Salmon R, Outbreak Control Team. Outbreak of verotoxin producing *E.coli* O157 infections involving over forty schools in south Wales, September 2005. Euro Surveill. 2005;10:e051006 1. **PMID: 16790877**

27. Whittaker PJ, Sopwith W, Quigley C, Gillespie I, Willshaw GA, Lycett C, et al. A national outbreak of verotoxin-producing *Escherichia coli* O157 associated with consumption of lemon-and-coriander chicken wraps from a supermarket chain. Epidemiol Infect. 2009;137:375–82. PubMed http://dx.doi.org/10.1017/S0950268808001702

28. Rechel B, Mahgoub H, Pritchard GC, Willshaw G, Williams C, Rodrigues B, et al. Investigation of a spatiotemporal cluster of verotoxin-producing *Escherichia coli* O157 infections in eastern England in 2007. Euro Surveill. 2011;16:19916. PubMed

29. Hart J, Smith G. Verocytotoxin-producing *Escherichia coli* O157 outbreak in Wrexham, North Wales, July 2009. Euro Surveill. 2009;14:19300. PubMed

30. Ihekweazu C, Carroll K, Adak B, Smith G, Pritchard GC, Gillespie IA, et al. Large outbreak of verocytotoxin-producing *Escherichia coli* O157 infection in visitors to a petting farm in South East England, 2009. Epidemiol Infect. 2012;140:1400–13. PubMed http://dx.doi.org/10.1017/S0950268811002111

31. Matulkova P, Gobin M, Taylor J, Oshin F, O'Connor K, Oliver I. Crab meat: a novel vehicle for *E. coli* O157 identified in an outbreak in South West England, August 2011. Epidemiol Infect. 2013;141:2043–50. PubMed http://dx.doi.org/10.1017/S0950268812002816

32. Launders N, Locking ME, Hanson M, Willshaw G, Charlett A, Salmon R, et al. A large Great Britain–wide outbreak of STEC O157 phage type 8 linked to handling of raw leeks and potatoes. Epidemiol Infect. 2016;144:1–18. **PMID: 26041509**
